# The Community-Level Interventions for Pre-eclampsia (CLIP) cluster randomised trials in Mozambique, Pakistan, and India: an individual participant-level meta-analysis

**DOI:** 10.1016/S0140-6736(20)31128-4

**Published:** 2020-08-22

**Authors:** Peter von Dadelszen, Zulfiqar A Bhutta, Sumedha Sharma, Jeffrey Bone, Joel Singer, Hubert Wong, Mrutyunjaya B Bellad, Shivaprasad S Goudar, Tang Lee, Jing Li, Ashalata A Mallapur, Khátia Munguambe, Beth A Payne, Rahat N Qureshi, Charfudin Sacoor, Esperança Sevene, Marianne Vidler, Laura A Magee, CLIP Trials Working Group, Eusébio Macete, Helena Boene, Felizarda Amose, Orvalho Augusto, Cassimo Bique, Ana Ilda Biz, Rogério Chiaú, Silvestre Cutana, Paulo Filimone, Emília Gonçálves, Marta Macamo, Salésio Macuacua, Sónia Maculuve, Ernesto Mandlate, Analisa Matavele, Sibone Mocumbi, Dulce Mulungo, Zefanias Nhamirre, Ariel Nhancolo, Cláudio Nkumbula, Vivalde Nobela, Rosa Pires, Corsino Tchavana, Anifa Vala, Faustino Vilanculo, Sana Sheikh, Zahra Hoodbhoy, Imran Ahmed, Amjad Hussain, Javed Memon, Farrukh Raza, Geetanjali M Katageri, Umesh S Charantimath, Shashidhar G Bannale, Keval S Chougala, Vaibhav B Dhamanekar, Narayan V Honnungar, Anjali M Joshi, Namdev A Kamble, Chandrappa C Karadiguddi, Avinash J Kavi, Gudadayya S Kengapur, Bhalachandra S Kodkany, Uday S Kudachi, Sphoorthi S Mastiholi, Geetanjali I Mungarwadi, Umesh Y Ramdurg, Amit P Revankar, Sharla K Drebit, Dustin T Dunsmuir, Chirag Kariya, Mansun Lui, Diane Sawchuck, Domena K Tu, Ugochi V Ukah, Mai-Lei Woo Kinshella, J Mark Ansermino, Ana Pilar Betrán, Richard Derman, Shafik Dharamsi, France Donnay, Guy Dumont, Susheela M Engelbrecht, Veronique Fillipi, Tabassum Firoz, William Grobman, Marian Knight, Ana Langer, Simon Lewin, Gwyneth Lewis, Craig Mitton, Nadine Schuurman, Andrew Shennan, Jim Thornton, Olalekan Adetoro, John O Sotunsa

**Affiliations:** a Department of Women and Children's Health, School of Life Course Sciences, Faculty of Life Sciences and Medicine, King's College London, London, UK; b Department of Obstetrics and Gynaecology, BC Children's Hospital Research Institute, University of British Columbia, Vancouver, BC, Canada; c Centre for Health Evaluation and Outcome Sciences, Providence Health Care Research Institute, University of British Columbia, Vancouver, BC, Canada; d Centre for Global Child Health, Hospital for Sick Children, Toronto, ON, Canada; e Centre of Excellence, Division of Woman and Child Health, Aga Khan University, Karachi, Pakistan; f KLE Academy of Higher Education and Research, Jawaharlal Nehru Medical College, Belagavi, Karnataka, India; g S Nijalingappa Medical College, Hanagal Shree Kumareshwar Hospital and Research Centre, Bagalkote, Karnataka, India; h Centro de Investigação em Saúde da Manhiça, Manhiça, Mozambique; i Department of Physiological Sciences, Clinical Pharmacology, Faculdade de Medicina, Universidade Eduardo Mondlane, Maputo, Mozambique

## Abstract

**Background:**

To overcome the three delays in triage, transport and treatment that underlie adverse pregnancy outcomes, we aimed to reduce all-cause adverse outcomes with community-level interventions targeting women with pregnancy hypertension in three low-income countries.

**Methods:**

In this individual participant-level meta-analysis, we de-identified and pooled data from the Community-Level Interventions for Pre-eclampsia (CLIP) cluster randomised controlled trials in Mozambique, Pakistan, and India, which were run in 2014–17. Consenting pregnant women, aged 12–49 years, were recruited in their homes. Clusters, defined by local administrative units, were randomly assigned (1:1) to intervention or control groups. The control groups continued local standard of care. The intervention comprised community engagement and existing community health worker-led mobile health-supported early detection, initial treatment, and hospital referral of women with hypertension. For this meta-analysis, as for the original studies, the primary outcome was a composite of maternal or perinatal outcome (either maternal, fetal, or neonatal death, or severe morbidity for the mother or baby), assessed by unmasked trial surveillance personnel. For this analysis, we included all consenting participants who were followed up with completed pregnancies at trial end. We analysed the outcome data with multilevel modelling and present data with the summary statistic of adjusted odds ratios (ORs) with 95% CIs (fixed effects for maternal age, parity, maternal education, and random effects for country and cluster). This meta-analysis is registered with PROSPERO, CRD42018102564.

**Findings:**

Overall, 44 clusters (69 330 pregnant women) were randomly assigned to intervention (22 clusters [36 008 pregnancies]) or control (22 clusters [33 322 pregnancies]) groups. 32 290 (89·7%) pregnancies in the intervention group and 29 698 (89·1%) in the control group were followed up successfully. Median maternal age of included women was 26 years (IQR 22–30). In the intervention clusters, 6990 group and 16 691 home-based community engagement sessions and 138 347 community health worker-led visits to 20 819 (57·8%) of 36 008 women (of whom 11 095 [53·3%] had a visit every 4 weeks) occurred. Blood pressure and dipstick proteinuria were assessed per protocol. Few women were eligible for methyldopa for severe hypertension (181 [1%] of 20 819) or intramuscular magnesium sulfate for pre-eclampsia (198 [1%]), of whom most accepted treatment (162 [89·5%] of 181 for severe hypertension and 133 [67·2%] of 198 for pre-eclampsia). 1255 (6%) were referred to a comprehensive emergency obstetric care facility, of whom 864 (82%) accepted the referral. The primary outcome was similar in the intervention (7871 [24%] of 32 290 pregnancies) and control clusters (6516 [22%] of 29 698; adjusted OR 1·17, 95% CI 0·90–1·51; p=0·24). No intervention-related serious adverse events occurred, and few adverse effects occurred after in-community treatment with methyldopa (one [2%] of 51; India only) and none occurred after in-community treatment with magnesium sulfate or during transport to facility.

**Interpretation:**

The CLIP intervention did not reduce adverse pregnancy outcomes. Future community-level interventions should expand the community health worker workforce, assess general (rather than condition-specific) messaging, and include health system strengthening.

**Funding:**

University of British Columbia, a grantee of the Bill & Melinda Gates Foundation.

## Introduction

Pregnancy hypertension (ie, all hypertensive disorders of pregnancy, including chronic hypertension, gestational hypertension, and pre-eclampsia) complicates approximately 10% of pregnancies in low-income and middle-income countries.^1^ Pre-eclampsia, the form of pregnancy hypertension associated with proteinuria or end-organ complications, or both, is associated with the greatest risk of maternal, fetal, and neonatal mortality and morbidity.^2^ Globally, pre-eclampsia is the second leading cause of direct maternal mortality, resulting in an estimated 76 000 maternal deaths and 500 000 fetal and newborn deaths every year.^3^ More than 99% of these deaths occur in low-income and middle-income countries, primarily in south Asia and sub-Saharan Africa.^4^

**Research in context**

**Evidence before this study**

We identified evidence from randomised controlled trials of the benefit of community mobilisation, task-sharing, and use of magnesium sulfate to improve pregnancy outcomes, and evidence from observational studies of the use of methyldopa in initial management of hypertensive emergencies in volume-restricted patients (eg, women with pre-eclampsia). The individual country Community-Level Interventions for Pre-eclampsia (CLIP) trials were done in India, Pakistan, and Mozambique between 2014 and 2017 to investigate pregnancy hypertension focused and solely community-level community engagement, antenatal assessment, risk stratification, initiation of life-saving therapies, and transport to hospital.

**Added value of this study**

The CLIP trials are the first to run a solely community-level intervention for pregnancy hypertension, designed to address the so-called three delays: triage, transport, and treatment. The existing community health workforce was scaled up to provide community engagement and community health worker-led app-guided triage, treatment, and transport to a facility for women with pregnancy hypertension. Overall, the CLIP intervention did not improve the primary composite of maternal, fetal, and newborn mortality and morbidity. Community health workers were able to undertake all aspects of the app-guided visits, and approximately 10% of pregnant women were found to be hypertensive using a device validated for use in pregnancy; however, few women were eligible for in-community treatment, and the numbers of community health workers were inadequate to reach all women who could have benefited. An increased number of app-guided visits was associated with improved outcomes, with substantial improvements from eight such contacts.

**Implications of all the available evidence**

Our findings suggest that community-level interventions addressing triage, initial treatment, and transport of women with pregnancy hypertension can be successfully completed by community health workers, but their numbers must be adequate to provide at least eight antenatal care contacts to reduce adverse outcomes. A focus on community-level intervention without facility enhancement is unlikely to yield the improvements in maternal and perinatal outcome as hoped or needed by the global health community. Further studies should assess a more comprehensive health strategy that involves facility strengthening and community-level activities restricted to accurate measurement of blood pressure and simple condition-specific interventions.

Generally, research in this field has focused on institutional-level interventions with magnesium sulfate (for eclampsia prevention and treatment) and parenteral antihypertensive therapy for severe hypertension.^4^ However, many women die or are irreversibly affected by pre-eclampsia because they never reach an inpatient facility, either because they die at home or on the way to a facility, or because they are already in a critical condition on arrival. As such, the concern is that if research continues to be restricted to inpatient, facility-level interventions, maternal lives will be lost due to pre-eclampsia and eclampsia from delays in triage, transport, and treatment (ie, the so-called three delays).^5^ A solution to these delays might be to provide care to women in their communities.

The Community-Level Interventions for Pre-eclampsia (CLIP) study (NCT01911494) comprised three cluster randomised controlled trials that aimed to reduce the burden of adverse maternal and perinatal outcomes related to pregnancy hypertension by addressing the three delays via community-level intervention. Trial design was informed by extensive feasibility studies between 2011 and 2014 with pregnant women, mothers, household proxy decision makers, health-care workers, traditional birth attendants and faith-based caregivers, community and opinion leaders, and health administrators. Literature and document reviews and qualitative, quantitative, and participatory methods were used to explore the societal context; acceptability of the intervention to women, community health workers (who delivered it), and health-care providers (who provided support, as required); government stakeholders (who would support implementation); and facility assessment of staff and resources (including commodities). Each of the three CLIP trials in India, Pakistan, and Mozambique, which were completed, was independently powered and designed a priori to contribute to an individual participant-level data meta-analysis that would improve power for assessment of maternal mortality or morbidity, and other components of the composite maternal and perinatal primary outcomes of the CLIP trials.^6^, ^7^, ^8^, ^9^

## Methods

### Study design

This individual participant-level meta-analysis included data from the CLIP cluster randomised controlled trials in India (run from November, 2014, to October, 2016), Pakistan (January, 2015, to December, 2016), and Mozambique (February, 2015, to February, 2017)^7^, ^8^, ^9^ as agreed by all CLIP principal investigators (ZAB, MBB, SSG, AAM, KM, RNQ, CS, ES) and detailed in the CLIP trial protocol. The included trials were cluster randomised controlled trials with 12 (India and Mozambique) or 20 clusters (Pakistan) comprising complex health system interventions.

The Individual Patient Data (IPD) proposal was prospectively registered with PROSPERO, CRD42018102564, as were the individual CLIP trials (NCT01911494; the protocol is in the appendix [pp 12–120]). Ethical approval for the trials and individual participant-level data meta-analysis was granted by the University of British Columbia, Vancouver, BC, Canada (H12-03497) and each country's relevant research ethics board (Aga Khan University, Pakistan, 2590-Obs-ERC-13; KLE University, India, MDC/IECHSR/2011-12/A-4, ICMR 5/7/859/12-RHN; and Centro de Investigação em Saúde da Manhiça [CIBS-CISM/038/14], Mozambique National Bioethic Committee [219/CNBS/14]). All eligible pregnant women provided written informed consent to participate.

### Study procedures

The units of randomisation (clusters) in India were primary health centres, in Pakistan were Union Councils (in Pakistan, provinces are divided into divisions, subdivisions [tehsil], and thereafter into Union Councils, comprising a large village and surrounding areas, often including nearby small villages) and all associated primary health centres, and in Mozambique were Administrative Posts (in Mozambique, provinces are divided into districts, which are further divided into Administrative Posts). Local teams chose potential clusters according to similar health-care infrastructure, accessibility for the surveillance team, and the absence of conflicting concurrent research activity. In India and Pakistan, internal pilot trials (each in an initial four clusters per country) preceded definitive trials. Pregnant women aged 15–49 years (12–49 years in Mozambique) were identified in their homes or local primary health centres by trained community health workers. Clusters were randomly assigned (1:1), via restricted, stratified randomisation according to population size, to either the intervention or control group. The trials were unmasked given the nature of the intervention. Participants in control clusters continued current local practice around antenatal care, referral to facilities, and initiation of therapy. The intervention aimed to address the so-called three delays in triage, transport, and treatment related to maternal mortality risk. The first step was community engagement involving women and their mothers, household male decision makers, mothers-in-law, and community leaders regarding pre-eclampsia awareness and education about birth preparedness and complication readiness, supported by culturally appropriate pictograms. Community group meetings were held in all countries, with additional one-on-one health-care worker-led meetings in women's homes in clusters in Pakistan. In the second step, community health workers were trained to task-share pregnancy-hypertension-oriented care at CLIP visits in women's homes, using the CLIP Pre-eclampsia Integrated Estimate of Risk (PIERS)-On-the-Move (POM) mHealth app for risk stratification.^10^ The team of community health workers already in place was trained to make the intervention scalable, if found to improve outcomes in a trial setting.^5^ Community health workers responded to emergency conditions (aided by country-specific pictograms), took women's blood pressure using the Microlife BP 3AS1-2 device (Widnau, Switzerland) and assessed dipstick proteinuria at the first contact and any subsequent contact when hypertension was detected. Per-protocol, the minimum number of CLIP visits should have been at least every 4 weeks, with additional visits recommended at days 3, 7, and 14 after birth. Community health workers were directed by the POM app to either administer oral methyldopa 750 mg for blood pressure of 160/110 mm Hg or higher; administer intramuscular magnesium sulfate 10 g for suspected severe pre-eclampsia (miniPIERS^11^ risk for an adverse maternal outcome of at least 25%, severe systolic hypertension [at least 160 mm Hg], eclampsia, stroke, or vaginal bleeding); or refer the woman to a comprehensive emergency obstetric care facility for suspected pre-eclampsia or increased risk of stillbirth (4+ dipstick proteinuria value, or absent fetal movements for at least 12 h). In referral facilities shared by both intervention and control groups, evidence-based care was promoted through a small number of continuous professional development events (three in India, three in Pakistan, and six in Mozambique) spaced throughout the trial periods that focused on the WHO's recommendations for prevention and treatment of pre-eclampsia and eclamspia.^12^

In intervention and control clusters, surveillance teams were trained to do regular cross-sectional surveys of households (every 3–6 months), except in India, where a prospective population-based surveillance system was established. After individual participant consent was obtained, data were collected on baseline individual-level and household-level information, antenatal care, and adverse maternal, fetal, and neonatal outcomes up to 6 weeks after birth (for the mother) or 28 days after birth (for the neonate). Women were defined as withdrawing from the trial if they declined further trial surveillance. Women were lost to follow-up if they were more than 6 weeks post partum (based on estimated delivery date) and more than one surveillance cycle from trial end. Women were defined as still on follow-up if they either had not delivered their baby or were post partum within 6 weeks of their estimated delivery date and within one surveillance cycle from the trial end. Overall coordination and data management was done by the Pre-eclampsia – Eclampsia Monitoring, Prevention and Treatment research group at The University of British Columbia.^7^, ^8^, ^9^

### Data analysis

We assessed the risk of bias of each trial according to the five criteria for cluster randomised controlled trials in the Cochrane Handbook: recruitment bias, baseline imbalance, loss of clusters, incorrect analysis, and comparability with individualised randomised trials.^13^

Within Research Electronic Data Capture (REDCap; version 5, Vanderbilt University, Nashville, TN, USA) we extracted data, by trial group, for characteristics of: the trial, women enrolled, and the intervention, and outcomes. For this meta-analysis, as for the individual trials, the primary outcome was one or more of the maternal or perinatal mortality or morbidity outcomes, and the secondary outcomes were birth preparedness and complication readiness, the proportion of births that occurred in a facility, and delivery in a facility that is able to provide comprehensive emergency obstetric care (panel). Secondary outcomes included components of the primary outcome (including a composite of maternal mortality or morbidity, and a composite of stillbirth, neonatal death, or neonatal morbidity) and safety outcomes. Adverse events that we assessed were transport-related injury or death and infection-site haematoma or infection after intramuscular magnesium sulfate in the community. In India (given trial surveillance informed by facility data), we also included as part of this meta-analysis maternal systolic blood pressure of below 110 mm Hg on arrival at a facility after in-community methyldopa; respiratory depression, coma, or death during transport after in-community magnesium sulfate; and infection-site haematoma or infection after intramuscular magnesium sulfate in-community or at a facility. We were unable to follow up the details of a woman's management and clinical courses after she was referred to facility. Serious adverse events were defined as being serious, unexpected (in nature, severity, or frequency), and thought to be related to the study intervention.

Panel **Outcome definitions for Community-Level Interventions for Pre-eclampsia trials and this meta-analysis**

**Primary outcome**

The primary outcome is a combined maternal or perinatal outcome (either maternal, fetal, or neonatal death, or severe morbidity for the mother or baby).

*Maternal outcomes*

Maternal death was defined as the number of deaths during pregnancy or within 42 days of pregnancy (or last contact day if contact was not maintained to 42 days) per 1000 identified pregnancies, defined as the maternal death rate.

Maternal morbidity was defined as the number of women with one or more life-threatening complication of pregnancy during pregnancy or within 42 days of pregnancy or last contact day if contact was not maintained to 42 day, per 1000 identified pregnancies. Complications of pre-eclampsia were defined as follows:

Serious end-organ complications of pre-eclampsia

• Eclampsia*:* occurrence of generalised convulsions during pregnancy, labour, or within 42 days of delivery in the absence of epilepsy or another condition predisposing to convulsions
• Stroke*:* hemiparesis, blindness, or both, developed during pregnancy or in the 42 days post partum, lasting longer than 48 h
• Coma*:* unconsciousness for ≥12 h
• Antepartum haemorrhage*:* vaginal bleeding of ≥15 mL with or without pain before the onset of labour
• Disseminated intravascular coagulation: abnormal bleeding from mucosa (mouth, ears, or both)

Other major causes of maternal mortality

• Obstetric sepsis*:* in the community, defined as fever and one of abdominal or uterine tenderness, foul smelling vaginal discharge or lochia, productive cough and shortness of breath, dysuria or flank pain, or headache and neck stiffness
• Vesicovaginal or rectovaginal fistula: continuous loss of urine, faeces, or both, after delivery

Life-saving interventions

• Cardiopulmonary resuscitation: a set of emergency procedures including chest compressions and lung ventilation applied in cardiac arrest victims
• Dialysis*:* haemodialysis, peritoneal dialysis, or both
• Mechanical ventilation (other than for caesarean section): intubation and ventilation not related to anaesthesia
• Blood transfusion*:* one or more units of blood product
• Interventions for major post-partum haemorrhage: brace sutures, external and internal uterine compression, antishock garment use, internal iliac artery ligation, or hysterectomy with or without transfusion

*Perinatal outcomes*

Perinatal and late neonatal death was defined as stillbirth (gestational age ≥20 weeks, ≥500 g, or both), early neonatal mortality (within 0–7 days of birth) and late neonatal mortality (within 8–28 days of birth) per 1000 identified pregnancies.

Neonatal morbidity was defined as occurrence of a primary neonatal morbidity during 0–28 days of birth per 1000 identified pregnancies. Primary neonatal morbidities were:

• Feeding difficulty: including inability to suckle normally or latch on to the mother's breast to feed even if the mother's milk is not let down
• Breathing difficulty: including grunting and in-drawing of the abdomen under the ribs
• Seizure: occurrence of any seizure event (fits)
• Lethargy: baby not appearing normally wakeful after activities such as feeding or sleeping
• Coma: a non-medically induced period of unconsciousness of any length
• Hypothermia: baby is cold to touch
• Umbilical cord infection: characterised by discharge from and redness around the umbilical stump
• Skin infection: any appearance of abnormally red, black, swollen, or blistered skin with pus
• Bleeding from anywhere on the body
• Jaundice**:** yellow skin and eyes
• CNS-related morbidity: abnormal amount of vomiting as defined by the parents or caregiver with bulging or sunken fontanelle

**Secondary outcomes**

Birth preparedness and complications readiness: defined as having completed three or more of arranging for transport, obtaining prior permission for transport, saving money for obstetric care, identifying a skilled birth attendant, and identifying a facility for birth.

In-facility birth, defined as any birth at a health-care facility.

Birth at a comprehensive emergency obstetric care facility, defined as birth at any centre that provides basic functions and capability of doing a caesarean section, giving safe blood transfusions, and provision of care for sick and low-birthweight neonates, including resuscitation.

For the primary individual participant-level data meta-analysis, we included all CLIP participants who were followed up with regards to the primary outcome of this meta-analysis. However, women who withdrew, were lost to follow-up, or still on follow-up at trial end were included in a sensitivity analysis in which we used the imputation from the primary trials in the individual participant-level data meta-analysis. Each trial was independent, no potential overlap existed between enrolled participants.

With a planned sample size of approximately 60 600 women in 44 clusters, we would have 80% power to find a 20% reduction in the composite maternal and perinatal outcome, from a baseline of 10·2% and with an intraclass correlation coefficient (ICC) of 0·006. Furthermore, this sample size would give similar power to find a 20% reduction in maternal mortality or morbidity if the baseline rate were 1·7% and the ICC were less than 0·001 (appendix pp 122–54). A 20% reduction was chosen a priori as being clinically relevant by consensus within the CLIP group of experts and the research programme technical advisory group.

We combined data from each trial dataset by study group. We summarised data as median (IQR) for continuous variables and n (%) for categorical variables. While in the primary trials we imputed outcomes for women with incomplete data, in this meta-analysis we only include data from women with complete follow-up.^7^, ^8^, ^9^ We assessed the treatment effect on the various outcomes using generalised mixed-effect models with random effects for both country and cluster, and fixed effects for the study group and baseline characteristics of maternal age, basic education, previous pregnancy (parity), and neonatal mortality rate from each country's baseline survey as part of the feasibility studies. We used a one-stage approach, in which a single model is fitted directly using the results from each study, to make optimal use of data.^14^ The summary statistic was the adjusted odds ratio (OR; fixed effect for maternal age, parity [nulliparous *vs* parous], and maternal education, and random effects for country and cluster) with Wald-type 95% CI. We assessed between-trial heterogeneity using the τ^2^ (estimated as the variance term of the random effect for treatment in the mixed-effect model) and *R*^2^ statistics (as the ratio of the SEs of the treatment effect from a model with fixed slope and a model with a random slope).^15^ A τ^2^ close to 0 and *R*^2^ close to 1 were taken as indicating a lack of heterogeneity. Statistical significance (two-sided) was set at a p value of less than 0·05 for the composite maternal and perinatal outcome, and a p value of less than 0·001 for other analyses.

We did four types of sensitivity analyses for the primary outcome and its components to assess the effect of potential sources of bias on our results. First, women who were defined as lost to follow-up or still on follow-up at the end of the trial were included to assess the potential effect of missingness. As for the primary analysis of the individual CLIP trials, mixed imputation was used to account for the risk associated with each woman, depending on her personal baseline characteristics, cluster characteristics, and time of enrolment relative to the beginning of the trial. Second, we restricted the adjusted analysis to women whose pregnancies continued to at least 20 weeks, because the average gestational age at recruitment was much earlier in India (approximately 11 weeks) than in Pakistan (approximately 21 weeks) and Mozambique (approximately 26 weeks).^7^, ^8^, ^9^ As for the individual CLIP trials and according to the statistical analysis plan (appendix pp 122–54), inclusion for this sensitivity analysis was: restricted to women who had 42 days of post-partum follow-up data; restricted to women with anticipated birth (according to estimated delivery date) or anticipated birth and 42 day of post-partum follow-up data within the trial timeline, to assess the effect of the intervention independent of gestational age at birth; and expanded to include women who were enrolled into trial surveillance only post partum, which might have reflected an effect of community engagement but was a protocol deviation. Third, we did an unadjusted analysis without accounting for baseline individual-level and cluster-level characteristics. Fourth, we did a so-called on-treatment analysis of women who received at least one community health worker-led POM-guided visit in intervention versus control clusters.

In an additional planned secondary analysis, we explored in the intervention group whether an association existed between our primary outcome and the number of CLIP visits, measured as 0, 1–3, 4–7, or 8 or more visits, to reflect previous and current WHO recommendations for the frequency of antenatal care contacts.^16^ To account for factors related to the number of POM-guided visits and confounders, the analysis was restricted to women whose pregnancies continued beyond 20 weeks and was adjusted for maternal age, basic education, parity, time of enrolment in the trial, and distance from the household to a facility.

We used R statistical software (version 3.5.2) for all analyses.

### Role of the funding source

The funder of the study had no role in study design, data collection, data analysis, data interpretation, or writing of the report. The corresponding author had full access to all data in the study and final responsibility for the decision to submit for publication.

## Results

The general characteristics of the CLIP trials, their participants, intervention implementation, and outcomes are presented in the appendix (pp 4–5).

In the three CLIP trials, comprising 44 clusters (n=22 intervention, n=22 control), 69 330 participants were enrolled (36 008 pregnancies in the intervention group and 33 322 in the control group), some of whom were pregnant more than once, such that we had a third more births than expected per cluster (appendix pp 11–121). 61 988 (89·4%) pregnancies were successfully followed up (32 290 [89·7%] in the intervention group and 29 698 [89·1%] in the control group), at similar and high rates across countries (appendix pp 4–5). Few women withdrew from the study (four [<0·1%] in the intervention group and five [<0·1%] in the control group). Loss to follow-up, which only occurred in Pakistan and Mozambique, was slightly lower (by 0·7%) in intervention clusters; loss to follow-up was attributed to migration and cyclical surveillance cycles by study site staff. Just under 8% of pregnancies (2811 [7·8%] in the intervention group and 2555 [7·7%] in the control group) were ongoing and still on follow-up on the trial end date.

The median maternal age of included women was 26 years (IQR 22–30) across all countries (appendix pp 4–5). Basic education, as measured by each country, had been received by over 60% of women in India (ie, at least 8 years of schooling) and Mozambique (ie, at least grade 5), but by no more than 20% in Pakistan (ie, at least 5 years of schooling). Overall, over 70% of women in all countries were parous. The median gestational age at trial enrolment was 19·0 weeks (IQR 12·5–26·8), with the earliest in India (approximately 11 weeks), followed by Pakistan (approximately 21 weeks) and Mozambique (approximately 27 weeks).

23 681 (6990 group and 16 691 home-based) community engagement sessions were held by the CLIP country teams (appendix pp 4–5). Training (of 2–15 days' duration) was delivered to 450 health-care providers, with refresher training every 1–6 months. Methyldopa and magnesium sulfate were sourced in-country. Trained community health workers provided 138 347 visits for a median of six visits per pregnancy, four (IQR two to six) antenatal and two (IQR one to three) post partum. 20 819 (57·8%) of 36 008 women in the intervention group had one or more POM-guided visits, approximately half of whom had visits that were compliant with the minimum frequency (11 095 [53·3%] of 20 819). Blood pressure was measured at almost all visits (137 705 [99·5%]), and dipstick proteinuria was done at 21 257 [96·4%] of 22 051 relevant visits as per the protocol (ie, first and all subsequent visits at which hypertension was detected). POM-guided visits resulted in recommendations at 136 755 (98·9%) visits. As previously published, hypertension was identified in 2111 [9·9%] of 21 306 pregnancies (636 [10·3%] of 6149 in India; 1010 [9·3%] of 10 904 in Pakistan; and 465 [10·9%] of 4253 in Mozambique).^1^ 181 (0·9%) of 20 819 women were eligible for treatment with oral methyldopa for severe hypertension and 198 (1·0%) were eligible for intramuscular magnesium sulfate for pre-eclampsia, and 1255 (6·0%) women were referred to a facility. Most women accepted the community health workers' POM-guided recommendations to administer oral methyldopa (162 [89·5%] of 181 eligible pregnancies) and intramuscular magnesium sulfate (133 [67·2%] of 198), and referral to a comprehensive emergency obstetric care facility (864 [81·6%] of 1255 pregnancies with recommended referral).

Protocol deviations occurred in few pregnancies (295 [0·9%] of 32 290) in the intervention group, of which 46 (15·6%) were related to treatment or referral not recommended as per the POM app and protocol (n=40 in India, n=4 in Pakistan, and n=2 in Mozambique) and 249 (84·4%) were antenatal CLIP visits by the community health workers among women only enrolled by the trial surveillance team during their post-partum period (n=6 in India, n=23 in Pakistan, and n=220 in Mozambique); pregnancies that had protocol deviations did not result in withdrawals or adverse events.

No effect of the CLIP intervention was seen on the primary composite maternal and perinatal outcome (7871 [24·4%] pregnancies in intervention group had one or more of the predefined maternal or perinatal mortality or morbidity outcomes *vs* 6516 [21·9%] pregnancies in the control group; adjusted OR 1·17, 95% CI 0·90–1·51) or its components, as was the case in each included cluster randomised controlled trial (table 1, figure).^7^, ^8^, ^9^ Most events were morbidities for the mother (6062 [9·8%] of 61 998 pregnancies) and neonate (6299 [10·2%]). Death was rare for mothers (143 [0·2%]), but not so for fetuses and neonates, with similar rates of stillbirth (2591 [4·2%]) and neonatal death (2677 [4·3%]). The ICC was 0.059. Similar patterns were seen across countries (appendix pp 4–5). The intervention had no effect on secondary outcomes (table 1). Approximately half of women in both groups showed birth preparedness and complication readiness. Most women delivered in a facility, with approximately half of women overall delivering in a comprehensive emergency obstetric care facility. We found no evidence of between-trial heterogeneity in outcomes. The gestational age at delivery was 39 weeks (IQR 37–41) for both intervention and control clusters.

**Table 1:** Primary and secondary outcomes

			**Event rate**		Adjusted odds ratio*	**p value**	**τ^2^**	**R^2^**
			Intervention clusters (n=32 290 women)	Control clusters (n=29 698 women)				
Primary composite maternal and perinatal outcome†			7871 (24·4%)	6516 (21·9%)	1·17 (0·90–1·51)	0·24	0·007	1·08
	Maternal outcome		3369 (10·4%)	2781 (9·4%)	1·20 (0·84–1·74)	0·32	0·003	1·01
		Maternal mortality	77 (0·2%)	66 (0·2%)	1·05 (0·67–1·64)	0·84	0·002	1·24
		Maternal morbidity	3319 (10·3%)	2743 (9·2%)	1·20 (0·83–1·74)	0·32	0·003	1·00
	Perinatal mortality, late neonatal death, or neonatal morbidity		5618 (17·4%)	4760 (16·0%)	1·10 (0·89–1·37)	0·38	0·003	1·06
		Stillbirth	1322 (4·1%)	1269 (4·3%)	1·03 (0·89–1·19)	0·69	0·003	1·00
		Neonatal mortality	1408 (4·4%)	1269 (4·3%)	1·10 (0·96–1·27)	0·17	0·005	1·44
		Neonatal morbidity	3463 (10·7%)	2836 (9·5%)	1·09 (0·73–1·62)	0·69	0·025	1·12
Secondary outcomes								
	Birth preparedness and complication readiness‡		15 875 (53·4%)	13 530 (45·5%)	0·91 (0·41–2·02)	0·82	0·001	1·01
	Proportion of facility births		25 397 (85·5%)	23 282 (78·7%)	1·06 (0·82–1·36)	0·66	0·01	1·13
	Birth at a comprehensive emergency obstetric care facility		14 657 (49·3%)	14 398 (48·5%)	0·84 (0·59–1·19)	0·32	0·001	1·01

Data are n (%) or adjusted odds ratio with 95% CI in parentheses, unless otherwise stated. τ^2^ is estimated as the variance term of the random effect for treatment in the mixed effect model and *R*^2^ is the ratio of the SEs of the treatment effect from a model with fixed slope and one with a random slope. CLIP=Community-Level Interventions for Pre-eclampsia.

* Adjusted for maternal age, parity, and maternal basic education.

† Defined as one or more of maternal morbidity or mortality, stillbirth, neonatal mortality, or neonatal morbidity; the primary outcome in the CLIP trials.

‡ Birth preparedness was defined as a “Yes” answer to all of the following: arranged for transport, obtained prior permission to seek emergency care, and saved money for obstetric care.

**Figure:**
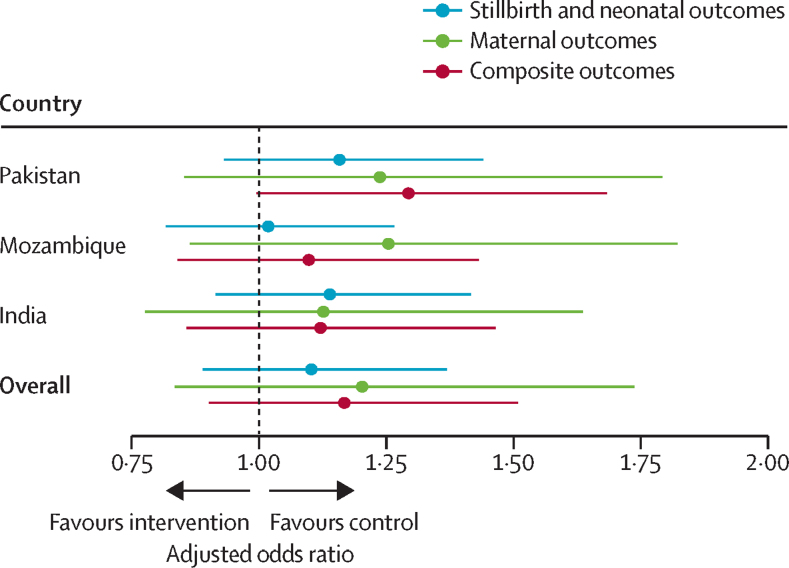
Forest plot of primary composite outcome and its components in each CLIP trial and overall Data points are adjusted odds ratios with 95% CIs indicated with whiskers. CLIP=Community-Level Interventions for Pre-eclampsia.

No serious adverse events related to the intervention or study occurred (appendix p 5) and few adverse events occurred. In one (2·0%) of 51 women in the intervention group in India, in-community receipt of methyldopa was associated with a decrease in blood pressure from a median of 167/108 mm Hg (IQR 162/100 to 178/113) to 160/100 mm Hg (146/90 to 170/110) after a median of 97 min (75–150). Injection-site haematoma or infection was reported only after receipt of magnesium sulfate in-facility (four [2·4%] of 168 in intervention group *vs* 1386 [3·5%] of 26 698 in the control group, in India only) and none was reported after in-community administration. No injuries related to transport to facility occurred.

Recruitment bias was regarded as low in all included trials. Although participants and their care providers were aware of their assignment to intervention or control clusters in this complex health system intervention study, enrolment in the trials was based on household residence. Clusters were defined by the unit of the primary health-care system associated with a woman's household; therefore, she could not simply choose to participate in another study area. Enrolment was slightly higher (by 8·7%), and loss to follow-up lower (by 0·7%) in intervention clusters than in control clusters (p<0·0001), which could cause concern about the risk of bias associated with baseline imbalances in the clusters. In India, one cluster was replaced by another with similar characteristics early in the pilot phase, and clearly before data analysis, in response to issues with primary health centre leadership and data integrity; otherwise, there were no concerns related to loss of clusters, incorrect analysis, or variability between clusters (because each trial accounted for clustering and reported ICC for each outcome).

In sensitivity analyses, the CLIP intervention had no effect when data for women defined as lost to or still on follow-up were imputed, unadjusted for baseline maternal characteristics, restricted to women whose pregnancies continued to at least 20 weeks, or in our on-treatment analysis restricted to women who received at least one community health worker-led POM-guided visit (adjusted OR 1·12, 95% CI 0·86–1·47, p=0·40; appendix p 6). However, there was an association between number of visits and outcomes (table 2). Fewer women with eight or more CLIP visits had a CLIP primary outcome or its components than those with no visits, an effect that was seen in each of the three countries (table 2; appendix p 8). The exception was neonatal morbidity, which was increased among those with eight or more visits (*vs* no visits) and not associated with a difference in gestational age at delivery (median 39 weeks in both intervention and control clusters; appendix p 9). Women with four to seven visits had an incidence of outcomes that were intermediate between those with eight or more visits and those with one to three visits. However, women with one to three visits had more adverse perinatal outcomes than did women without a POM-guided visit; we have previously reported that these women were enrolled 2–4 weeks earlier than those without a POM-guided visit, but we did not identify other baseline differences.^7^, ^8^, ^9^

**Table 2:** Sensitivity analysis of the association between number of community health-care worker POM-guided CLIP visits and the primary outcome among 30 721 pregnancies in CLIP*

		**0 visits**		**1–3 visits**			**4–7 visits**			**≥8 visits**		
		Event rate (n=10 754)	Adjusted odds ratio†	Event rate (n=4309)	Adjusted odds ratio†	p value	Event rate (n=8974)	Adjusted odds ratio†	p value	Event rate (n=6684)	Adjusted odds ratio†	p value
Primary outcome‡		2928 (27·2%)	1 (ref)	1251 (29·0%)	1·07 (0·98–1·18)	0·16	2340 (26·1%)	0·95 (0·88–1·03)	0·21	1242 (18·6%)	0·88 (0·80–0·97)	0·0079
Maternal outcome		1246 (11·6%)	1 (ref)	539 (12·5%)	0·96 (0.84–1·08)	0·49	1040 (11·6%)	0·92 (0·82–1·02)	0·11	436 (6·5%)	0·86 (0·74–0·99)	0·032
	Maternal mortality	24 (0·2%)	1 (ref)	11 (0·3%)	0·96 (0·42–2·18)	0·92	14 (0·2%)	0·57 (0·28–1·19)	0·14	5 (0·1%)	0·32 (0·11–0·92)	0·035
	Maternal morbidity	1229 (11·5%)	1 (ref)	535 (12·4%)	0·97 (0·86–1·10)	0·63	1032 (11·5%)	0·93 (0·83–1·03)	0·16	435 (6·5%)	0·87 (0·76–1·01)	0·060
Fetal or neonatal adverse outcome		2132 (19·9%)	1 (ref)	922 (21·5%)	1·16 (1·05–1·28)	0·0047	1634 (18·2%)	0·96 (0·88–1·05)	0·37	928 (13·9%)	0·86 (0·77–0·95)	0·0037
	Stillbirth	505 (4·7%)	1 (ref)	279 (6·5%)	1·54 (1·30–1·81)	<0·0001	396 (4·4%)	0·88 (0·76–1.02)	0·098	142 (2·1%)	0·41 (0·33–0·51)	<0·0001
	Neonatal mortality	545 (5·3%)	1 (ref)	260 (6·5%)	1·34 (1·14–1·58)	0·0005	414 (4·8%)	0·93 (0·81–1·08)	0·35	188 (2·9%)	0·66 (0·54–0·81)	<0·0001
	Neonatal morbidity	1266 (11·8%)	1 (ref)	485 (11·3%)	0·87 (0·76–0·99)	0·034	986 (11·0%)	0·99 (0·89–1·10)	0·79	725 (10·9%)	1·24 (1·09–1·41)	0·0016

Data are number of events and proportion of total events by visit number category, and adjusted odds ratio with 95% CI in parentheses, unless otherwise stated. CLIP=Community-Level Interventions for Pre-eclampsia. POM=Pre-eclampsia integrated estimate of risk-On-the-Move.

* These analyses included the 30 721 women in intervention clusters who were followed-up, excluding the 1341 women who were followed-up (ie, excluding 1341 women from the total population who were recruited and had a miscarriage before 20 weeks).

† Adjusted for maternal characteristics (as in the primary analysis: maternal age, parity, basic education, and gestational age at enrolment; enrolment timing in the trial; and distance from the household to facility.

‡ Defined as one or more of maternal morbidity or mortality, stillbirth, neonatal mortality, or neonatal morbidity; the primary outcome in the CLIP Trials.

## Discussion

The three CLIP cluster randomised controlled trials in India, Pakistan, and Mozambique involved just over 60 000 pregnancies in 44 clusters. 23 681 community engagement sessions were run, in groups in all countries and one-on-one with women and their immediate community in Pakistan. Community health workers proficiently carried out POM-guided visits for pregnant women in their communities, successfully measuring blood pressure and proteinuria, and providing POM app-guided triage and initial treatment of severe hypertension (with methyldopa) and suspected severe pre-eclampsia (with magnesium sulfate). Nevertheless, our community-level intervention for pregnancy hypertension had no effect on a composite of all-cause maternal and perinatal mortality or morbidity compared with care as usual, as observed in the individual country-level trials.^7^, ^8^, ^9^

Our findings are unlikely to be related to low statistical power, despite our higher than anticipated ICC (ie, 0·059 *vs* 0·006 anticipated). The event rate (14 387 [23·2%] of 61 988 pregnancies) was higher than anticipated (10·2%), as it was for the combined maternal outcome of maternal mortality and morbidity (ie, 6150 [9·9%] of pregnancies *vs* 1·7% anticipated). Also, the 95% CI of the adjusted OR of the combined outcome and its maternal, fetal, and neonatal components did not include the 20% reduction considered a priori to be important.

The CLIP intervention did not reach all women who could have benefited from it. First, only about two-thirds of women enrolled in intervention clusters received at least one CLIP visit, and just over half of these women had visits at the target frequency of once every 4 weeks. This limitation in human resource capacity in study areas came from our decision to use the existing community health worker workforce for scalability. However, expansion of this workforce might be worthwhile given that a higher number of POM-guided CLIP visits reduced the odds of the composite primary outcome and its components, other than survivable neonatal morbidity, which increased. This benefit was seen with eight or more visits, consistent with the WHO recommendations for eight antenatal care contacts.^16^ Second, the POM-guided care algorithm focused on referrals for systolic hypertension, detected in 1255 visits (0·9% of 138 347 visits; 6·0% of 20 819 pregnancies). This approach was taken to direct resources to women at highest risk of stroke and not overwhelm referral facilities, and to enable measurement of blood pressure by palpation (using an inflatable cuff) given inadequate access to accurate and affordable equipment in our study settings. However, in this young maternal population, isolated diastolic hypertension predominated (1314 [62%] of 2111 women with hypertension),^1^ and the blood pressure device used in CLIP has proven to be both usable and affordable.^17^ Whether referral of more women with hypertension to the relevant facilities would have been possible without overwhelming their scarce resources, or whether community-level treatment of hypertension could have improved outcomes is unknown.

Despite the importance of hypertension as a cause of maternal and perinatal mortality and morbidity, treatment of severe hypertension and use of magnesium sulfate for eclampsia prevention were aspects of the CLIP intervention for which only a small proportion of women were eligible. Despite the evidence base for effectiveness and safety of these treatments^18^, ^19^ and shown safety in our study settings, the rarity that women were eligible to receive them makes the likelihood that these treatments would have had an effect on outcomes overall is low. Notably, considerable training and permissions for task-sharing are required for community health workers to administer intramuscular magnesium sulfate. Although oral methyldopa for severe hypertension was indicated for a similarly small proportion of women, administration was straightforward, which raises the potential for community-level treatment of low-risk hypertension (ascertained by miniPIERS risk prediction^11^) and managed by a simple antihypertensive algorithm.^20^ By contrast, blood pressure measurement, using the Microlife BP 3AS1-2 device validated for use in pregnancy, showed that at least 10% of women in our study settings had pregnancy hypertension that was usually gestational hypertension without proteinuria, a condition amenable to enhanced surveillance and timed delivery.^1^

This individual participant-level meta-analysis is a novel meta-analysis of trials testing a combination of community engagement and mHealth-supported task-sharing with community health workers focused on pregnancy hypertension. In a 2015 Cochrane review of community-based interventions in pregnancy,^21^ eight of 26 cluster randomised controlled trials included studied a package of community mobilisation and home visits. Six of these cluster randomised controlled trials were in India or Pakistan. Three cluster randomised controlled trials focused on home counselling and education^22^, ^23^ or home visits by traditional birth attendants who inquired about warning symptoms of pregnancy complications.^24^ However, five cluster randomised controlled trials focused on newborn interventions^25^, ^26^, ^27^, ^28^, ^29^ and five focused on strengthened labour and delivery care, with or without provision of clean delivery kits.^24^, ^25^, ^27^, ^28^, ^29^, ^30^ Community-based intervention packages were associated with a non-significant decrease in maternal mortality and significant decreases in maternal morbidity, stillbirth, and neonatal mortality; neonatal morbidity was not reported.^21^ The decrease in maternal morbidity was driven by results of one trial in Pakistan where the traditional birth attendants received clean delivery kits and strengthened the provision of facility care in the intervention group.^24^ By contrast, the CLIP trials used community-level hypertension-focused antenatal care, with minimal facility-enhanced quality of care in both intervention and control groups. With regards to the reduction in stillbirth, two of three trials provided broad additional antenatal care training to community health workers who also worked with traditional birth attendants to enhance the quality of labour and delivery care.^27^, ^28^ CLIP focused only on pregnancy hypertension-related antenatal care with minimal facility enhancement in all clusters. Finally, five of six trials that reported neonatal mortality had newborn care as part of the intervention.^25^, ^27^, ^28^, ^29^, ^30^ CLIP focused entirely on community-level interventions for the mother and fetus, but not for the neonate.

Strengths of our analysis include the planned meta-analysis and harmonisation of study processes and outcome definitions; the large size of the trial populations and comprehensive recruitment of almost all eligible unselected pregnant women to mirror routine antenatal care; successful implementation of community health worker-led POM-guided antenatal care visits oriented around hypertension in women's community with regards to reach and fidelity; that the intervention and outcome assessment were done by different teams; and the consistency of findings across three countries. Limitations include potential ascertainment bias, which cluster randomised controlled trials are susceptible to; nevertheless, any difference in recruitment and loss to follow-up in intervention clusters was very small. Another limitation is our reliance on the existing community health-care workforce, which affected the extent to which the intervention could be delivered. Although designed to reflect the scalability and sustainability of the intervention, our study has emphasised that the health systems at our study sites are currently inadequate to implement a complex, condition-specific, community-level intervention. A focus on the community without facility enhancement was an additional limitation, but we have gathered unique information about the merits of community-level intervention in isolation. We were unable to assess the effect of the CLIP visit content on outcomes. Strengths of the CLIP visit content were that blood pressure and proteinuria were measured at almost all relevant visits and that community health workers were proficient with the POM app; however, the number of women who were eligible to receive oral methyldopa or intramuscular magnesium sulfate was too small to make an analysis of acceptance of in-community treatment feasible. A full process evaluation is planned to explore more comprehensively the context (including cost), implementation, and mechanisms of effect of our intervention in each of the three CLIP countries.^31^

Management and detection of pregnancy hypertension provide a gateway to excellence in maternity care. Ideally a health system should be able to identify and respond to pregnancy hypertension, induce labour, and provide both safe caesarean deliveries and care in the post-partum period. Achievement of these markers of good quality maternity care would contribute substantially to achievement of Sustainable Development Goal 3.1.

In summary, our findings suggest that community-level intervention addressing triage, initial treatment, and transport of women with pregnancy hypertension can be successfully implemented by community health workers, but their numbers must be adequate to provide at least eight antenatal care contacts to reduce adverse outcomes. Even then, the reduction is not large, suggesting that a focus only on community-level intervention without facility enhancement is unlikely to yield the improvements in maternal and perinatal outcome as hoped or as needed by the global health community. Further study should include community-level care restricted to adequate measurement of blood pressure and simple condition-specific interventions as part of a comprehensive health-strengthening programme.

## Data sharing

A data sharing statement for the CLIP trials is in the appendix (pp 150–51).
