# Department of Error

**DOI:** 10.1016/S0140-6736(20)31926-7

**Published:** 2020-09-19

*von Dadelszen P, Bhutta ZA, Sharma S, et al. The Community-Level Interventions for Pre-eclampsia (CLIP) cluster randomised trials in Mozambique, Pakistan, and India: an individual participant-level meta-analysis.* Lancet *2020; **396:** 553–63*—The appendix of this Article has been corrected as of Sept 17, 2020.

